# ﻿Re-establishment of *Sileneneglecta* Ten. (Caryophyllaceae) with taxonomic notes on some related taxa

**DOI:** 10.3897/phytokeys.195.81370

**Published:** 2022-05-11

**Authors:** Llorenç Sáez, Melilia Mesbah, Javier López-Alvarado, Gianluigi Bacchetta, Ridha El Mokni, Lorenzo Peruzzi, Bengt Oxelman

**Affiliations:** 1 Systematics and Evolution of Vascular Plants (UAB) – Associated Unit to CSIC, Dept. BABVE, Autonomous University of Barcelona, ES08193 Bellaterra, Spain Autonomous University of Barcelona Bellaterra Spain; 2 Gothenburg Global Biodiversity Centre, Box 461, 405 30 Gothenburg, Sweden Gothenburg Global Biodiversity Centre Gothenburg Sweden; 3 Laboratory of Ecology and Environment, Faculty of Natural and Life Sciences, University of Bejaia, 06000 Bejaia, Algeria University of Bejaia Bejaia Algeria; 4 Centre for Conservation of Biodiversity (CCB), Department of Life and Environmental Sciences, University of Cagliari, Cagliari, Italy University of Cagliari Cagliari Italy; 5 University of Monastir, Department of Pharmaceutical Sciences ‘A’, Laboratory of Botany, Cryptogamy and Plant Biology, Faculty of Pharmacy of Monastir, av. Avicenna, TN-5000 Monastir, Tunisia University of Monastir Monastir Tunisia; 6 Department of Biology, University of Pisa, Pisa, Italy University of Pisa Pisa Italy; 7 Department of Biological and Environmental Sciences, University of Gothenburg, Carl Skottsbergs Gata 22 B, 413 19 Gothenburg, Sweden University of Gothenburg Gothenburg Sweden

**Keywords:** Mediterranean Basin, morphometrics, nomenclature, taxonomy

## Abstract

*Sileneneglecta* has been misunderstood and confused with *S.nocturna*, although several morphological characters (petal shape, calyx indumentum, hairiness of stamen filaments, seed size, seed-coat surface and shape) allow separation of these species. Moreover, *S.mutabilis* (which has been considered conspecific with *S.neglecta*) and *S.martinolii* (an alleged endemic species to south-western Sardinia) are considered here as taxonomic synonyms of *S.nocturna* and *S.neglecta*, respectively. These taxonomic conclusions are strongly supported by multivariate morphometric analyses of 21 characters.

## ﻿Introduction

*Silene* L. is one of the large genera in *Caryophyllaceae*, comprising around 850 taxonomic species (Jafari et al. 2020). The genus is widely distributed in temperate regions mainly in the Northern Hemisphere, with the centre of its diversity is in western Asia and the Mediterranean Basin.

Silenesect.Silene is one of the largest sections of the genus, as classified by Jafari et al. (2020). It comprises about 93 species mainly distributed in the Mediterranean. It is characterised by monochasial (sometimes dichasial) inflorescence, usually non-auriculate petal claws and often excavate or flat seeds with long and narrow testa cells (Jafari et al. 2020). *Silenenocturna* L., *S.neglecta* Ten. and *S.mutabilis* L. are annual species grouped in this section (Jafari et al. 2020; Mesbah et al., in prep.).

Tenore (1826) described *Sileneneglecta* from southern Italy, whereas twelve years later (Tenore 1838), illustrations of the species which revealed its key characteristics were published. The taxon has been recognised at different levels, either as a separate species (Maire 1963; Pignatti 1982, 2017; Talavera 1990) or at subspecies level within *S.nocturna* (Arcangeli 1882; Fiori and Paoletti 1896; Chater et al. 1993). In a taxonomic study of the *S.nocturna* complex in Italy, Peruzzi and Carta (2013) proposed the species rank for *S.neglecta*, by providing morphological and karyological data and typifying the name. Later, in a study of original materials of some Linnaean names currently included within *Silene*, Peruzzi et al. (2014) concluded that the first available name at species level for the plants called *S.neglecta* was *S.mutabilis*. However, the application of the name *S.mutabilis* remains uncertain at present, since some authors have continued to use the name *S.neglecta* (Pignatti 2017; Bosch et al. 2019). Furthermore, *S.martinolii* Bocchieri & B.Mulas, an alleged endemic species to islets of south-western Sardinia, is morphologically very close to *S.neglecta* (Bocchieri and Mulas 1988), so it is advisable to clarify its taxonomic position.

This study aims to provide distinction between these taxa (*Sileneneglecta*, *S.nocturna*, *S.martinolii* and *S.mutabilis*), based on macromorphological features and Scanning Electron Microscopy (SEM) observations of hairs and seeds.

## ﻿Material and methods

### ﻿Plant material

The present morphological and comparative study is based on the examination of specimens in the field and in herbarium/laboratory and on the analysis of the protologues. The names of the specimens were applied *a priori* following Bocchieri and Mulas (1988) and Pignatti (2017) for *Silenemartinolii*; Linnaeus (1753) for *S.mutabilis*; Talavera (1990) and Pignatti (2017) for *S.neglecta* and Talavera (1990), Peruzzi and Carta (2013) and Pignatti (2017) for *S.nocturna*. Morphological characters, recognised as taxonomically discriminant within the *Silenenocturna* complex (e.g. Talavera 1990; Peruzzi and Carta 2013; Bacchetta et al. 2014; Peruzzi et al. 2014 and our own observations), were scored either in the field or in the herbarium specimens (BC, BCN, CAG, GB, ENSA, HJBS, JACA, LINN, MA, NAP, PI and WAG; acronyms according to Thiers (2022 [continuously updated]). Digital images from online databases for the Herbaria MPU, K, P and US were also examined. Morphological observations of materials were carried out under a binocular stereoscopic microscope Zeiss Stemi DV4 with eyepiece micrometer. Micromorphology was observed on calyces and mature seeds which were glued directly to aluminium stubs, coated with 40–50 nm gold and examined with a scanning electron microscopy (Hitachi 2300-SEM) at 20 kV. Given that detailed information on the seeds of *S.nocturna* has been provided in recent works (Peruzzi and Carta 2013; Peruzzi et al. 2014), seed data, based on SEM, provided for this species refers only to specimens that were misidentified as *S.neglecta* or *S.mutabilis*.

### ﻿Data analysis

A total of 21 characters were selected and scored in 71 specimens. From the total 21 morphological characters, 15 were quantitative, three were calculated ratios and three were qualitative (Table 1). A non-metric multidimensional scaling (NMDS; Kruskal 1964), which represents the relationships amongst individuals in a reduced dimension scatterplot and Cluster Analysis (CA) using the average linkage method (UPGMA; Michener and Sokal 1957), which allows the classification of individuals by similarity, were performed with PAST 4.08 (Hammer et al. 2001). The similarity matrix was calculated using the Gower coefficient, suitable for mixed data (Gower 1971).

**Table 1.** Morphological variables used in morphometric analyses.

Abbreviation	Character name	Type
RL/W	Ratio length/width of longest stem leaf	Calculated ratio
EHLS	Length of longest eglandular hair on lower part of stem (mm)	Quantitative
EHMS	Length of longest eglandular hair on middle part of stem (mm)	Quantitative
GHMS	Length of longest glandular hair on middle part of stem (mm)	Quantitative
EHI	Length of longest eglandular hairs on inflorescence axis (mm)	Quantitative
GHI	Length of longest glandular hairs on inflorescence axis (mm)	Quantitative
RF/cm	Ratio number of flowers/cm	Calculated ratio
CLLF	Length of calyx of lowest flower (mm)	Quantitative
TL	Length of longest calyx tooth (mm)	Quantitative
TW	Width of longest calyx tooth (mm)	Quantitative
RCL/TL	Ratio calyx length/ calyx tooth length	Calculated ratio
EHCL	Length of longest eglandular hair between calyx veins (mm)^1^	Quantitative
GHCL	Length of longest glandular hair between calyx veins (mm)^1^	Quantitative
EHCLV	Length of longest eglandular hair on calyx veins (mm)^1^	Quantitative
GHCLV	Length of longest glandular hairs on calyx veins (mm)^1^	Quantitative
MHC	Main type of hairs on calyx^1^	Qualitative
PL	Petal limb incision^2^	Qualitative
SH	Stamen filament pubescence^3^	Qualitative
GL	Gonophore length (mm)	Quantitative
SL	Largest seed length (mm)	Quantitative
SW	Largest seed width (mm)	Quantitative

^1^0: mainly eglandular; 1: approximately equal amounts of eglandular and glandular hairs; 2: mainly glandular. ^2^1: incision > 50% of limb length; 0 < 50% length of limb length. ^3^0: all stamens glabrous; 1: 50% stamens hairy; 2: all stamens hairy.

## ﻿Results and discussion

The variation, based on morphometric analysis (NMDS and CA) and the morphological characters of *Sileneneglecta*, *S.nocturna*, *S.martinolii* and *S.mutabilis*, are described and the taxonomic value of the characters is discussed.

### ﻿Morphometric analysis

**Figure 1.**
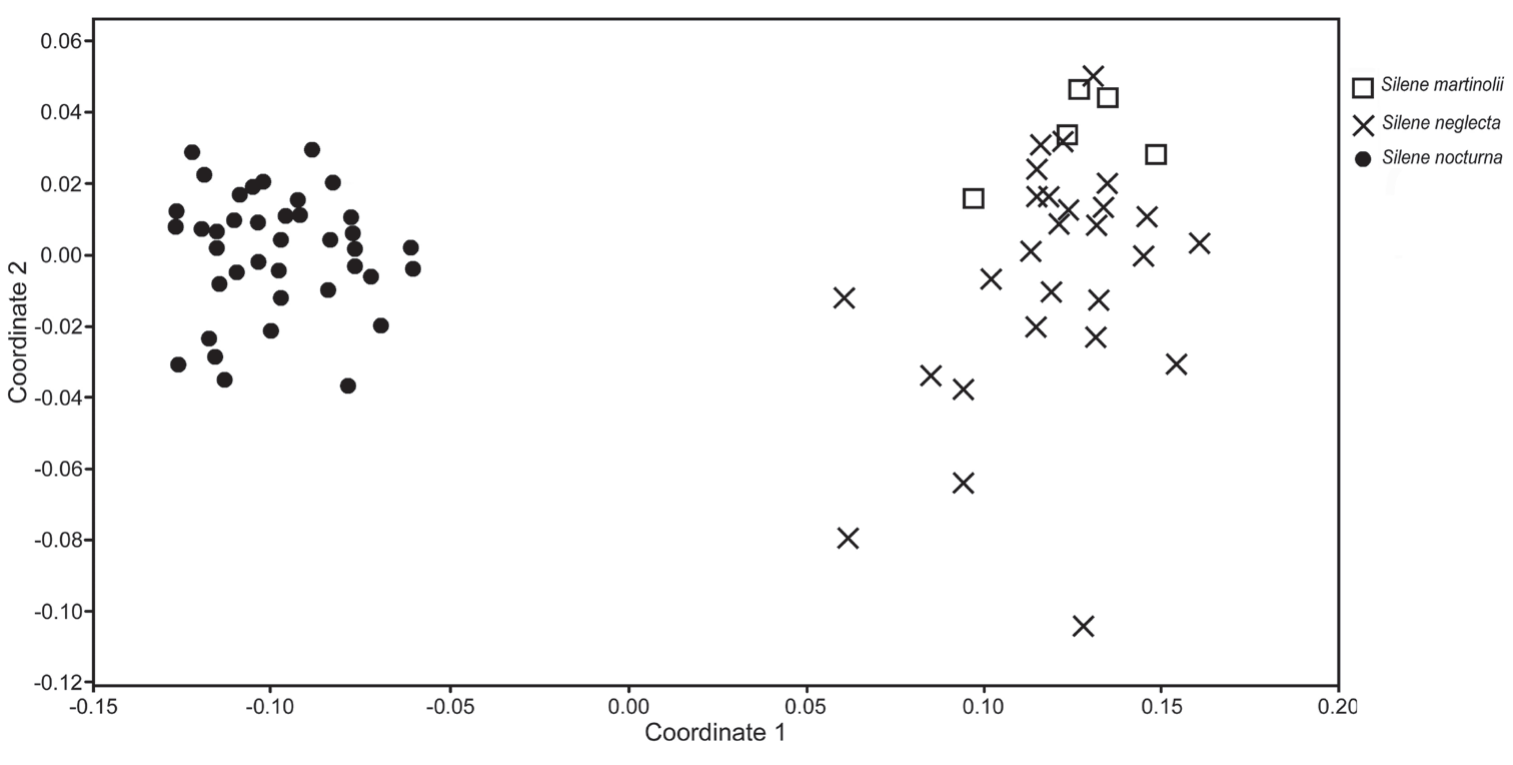
Non-metric multidimensional scaling scatterplot showing the first two dimensions of the analysis.

**Figure 2.**
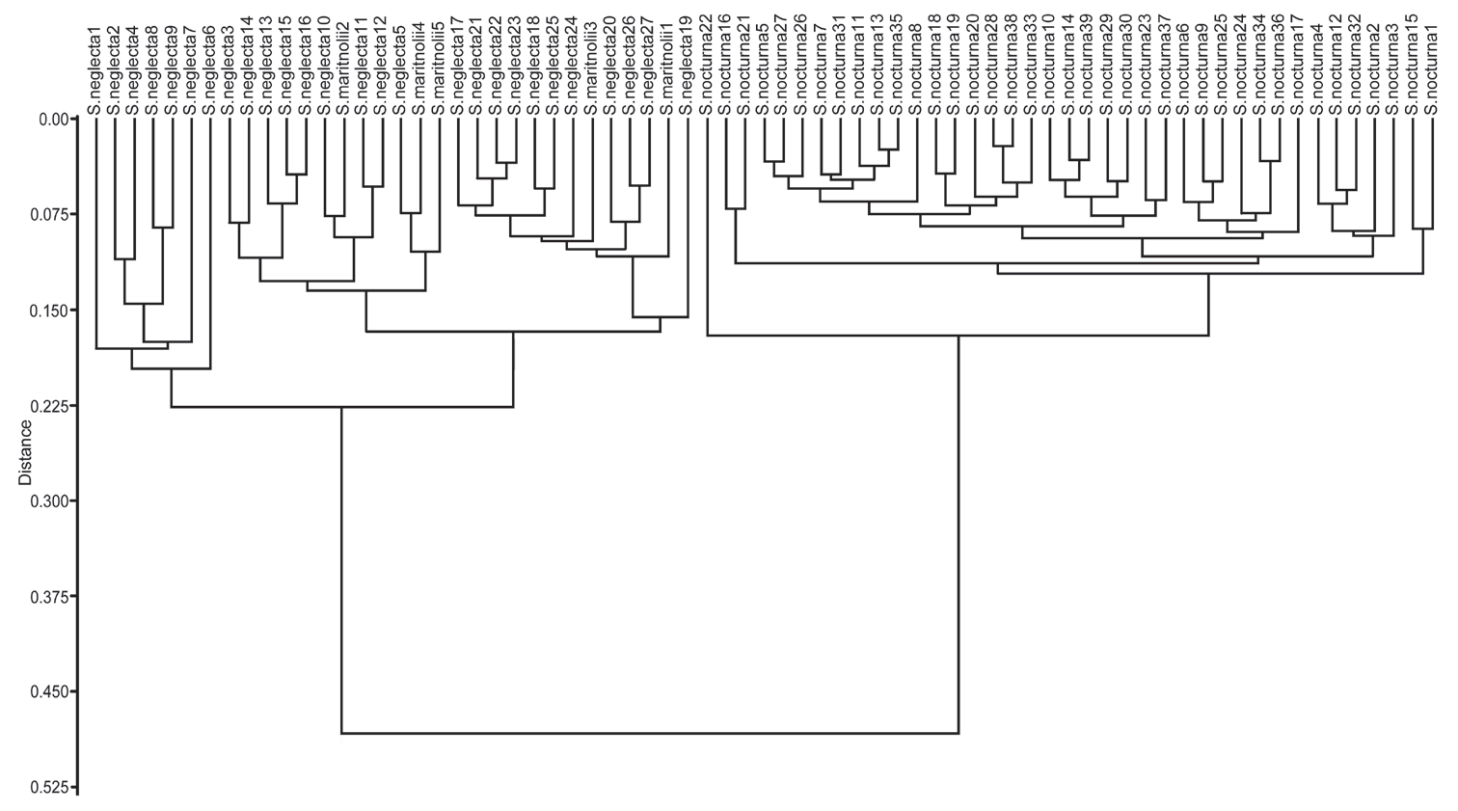
Hierarchical clustering of individuals of *Silenemartinolii*, *S.neglecta* and *S.nocturna* using paired group algorithm (UPGMA) and Gower Similarity Index.

The NMDS, performed with three dimensions, yielded a value of stress of 0.10 corresponding to a good ordination result (Clarke 1993). The scatterplot showed two clearly defined groups, where *Silenemartinolii* is intermingled with *S.neglecta* and both are separated from *S.nocturna* (Fig. 1). The UPGMA dendogram (Fig. 2) yielded two well-defined clusters, one formed by individuals of *S.neglecta* and *S.martinolii* and a second one formed exclusively by individuals of *S.nocturna*. The cophenetic correlation coefficient was 0.98, indicating a good fit between the cophenetic value matrix and the similarity matrix.

### ﻿Comparative morphology

#### Habit and hairiness

All the studied species are annuals, except some specimens of *Sileneneglecta* from maritime sands in north-western Tunisia (Tabarka) which are biennials (monocarpic forming a leaf rosette in the first year). Concerning all the other characters studied (and also molecular data, Mesbah et al., in prep.), these Tunisian plants are perfectly identified as *S.neglecta*. Close to Tabarka, populations of typical annual plants of *S.neglecta* can be found. The middle and upper parts of the stems of *S.neglecta* and *S.martinolii* are usually densely covered by glandular hairs up to 1.3 mm long, intermixed with eglandular hairs up to 1.9 mm long, whereas in *S.nocturna* (including the lectotype of *S.mutabilis*), the glandular hairs are up to 0.3 mm long. Peruzzi & Carta (2013) documented some differences in the hairiness of the basal portion of the stem between *S.neglecta* and *S.nocturna* (hairs longer than 1 mm in *S.neglecta* vs. hairs less than 1 mm long in *S.nocturna*). However, our observations indicate that individuals of *S.nocturna* with hairs longer than 1 mm long (up to 2.5 mm long) in the basal portion of the stem are not rare [Algeria: Crête Rouge (ENSA); Batna (GB); Croatia: Paklenica (PI); France: Garlaban (BC); Spain: Villareal (BC), Maresme (BC), Hospitalet (BC)].

#### Leaves

Leaf outline in *Sileneneglecta*, *S.martinolii* and *S.nocturna* varies from spatulate to linear, usually tapering more or less progressively from the base towards the inflorescence. *Sileneneglecta* has usually wider leaves than *S.nocturna*, but there is a notable variability with respect to this character so that it cannot be used for identification purposes. *Silenenocturna* sometimes has narrowly linear leaves in the middle and upper part of the stem (e.g. the lectotype of *S.mutabilis*), whereas in *S.neglecta*, they are spatulate to lanceolate or linear-lanceolate.

#### Pedicels

**Figure 3.**
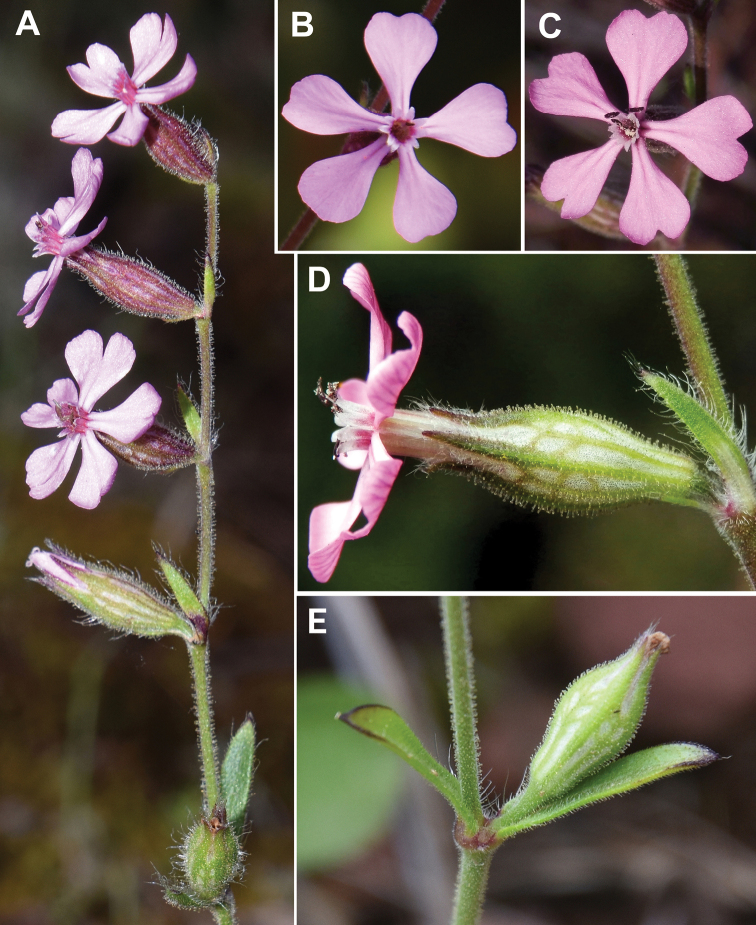
Field photos of *Sileneneglecta***A** inflorescence **B** flower with subentire petals **C** flower with emarginate petals **D** flower in lateral view **E** lowermost pedicel. Spain, Gavà, Roca del Barret, 29 Apr 2021, *L. Sáez* (herb. pers.).

The pedicels are accrescent and their length is somewhat variable within a single taxon. Our study reveals that this character presents much more variability than has been documented so far. The presence of long pedicels has been attributed to *Sileneneglecta* (Peruzzi and Carta 2013) and, based on this character, *S.neglecta* and *S.mutabilis* were later considered as synonyms (Peruzzi et al. 2014). Indeed, *S.neglecta* can have remarkably long lowermost pedicels, up to twice the length of the calyx (Italy, MPU300592). However, there are specimens of *S.neglecta* with lowermost pedicels equal to or shorter than the calyx [Algeria: Kabylie de Collo (P), Bône (P); Spain: Roca del Barret (LS7707), Tunisia: Tabarka (L. Sáez, herb. pers.)] (Fig. 3) and specimens of *S.nocturna* with pedicels longer (up to 22 mm long) than calyx [Spain: Sant Julià (BC), Formentera (BC), Camí Geganta (BC), Hospitalet (BC), Unzue (BC), Lluc (HJBS); Tunisia: Melloula, Tabarka, Bizerta (L. Sáez, herb. pers.)]. The pedicels of *S.martinolii* are usually shorter than to equal to the calyx length (Bocchieri and Mulas 1988), although in some specimens (Sa Corona su Crabi, CAG), the pedicel is longer than the calyx, even without being the lowermost flower. Therefore, this character cannot be used for taxonomic purposes.

The inclination of the lowest flower’s pedicel (in fruiting period) has been used by Peruzzi and Carta (2013) as a character to separate *Sileneneglecta* (up to 90°) from *S.nocturna* (up to 40°). Although this relatively strong inclination is certainly observed in some specimens of *S.neglecta* (including the lectotype), there are also several specimens of this species with the lowest flower’s pedicel suberect or erect-patent (Spain: LS7707, Fig. 3; Tunisia: Tabarka). Bocchieri and Mulas (1988) attributed erect-patent pedicels to *S.martinolii*. Although this is true in most cases, in two specimens (S’Aquasa Canna and Sa Corona su Crabi, CAG), several clearly patent lowest flower’s pedicels (ca. 90°) are observed.

#### Gonophore

Both species have puberulent gonophores, being longer in *Sileneneglecta* and *S.martinolii* than in *S.nocturna* (Table 2). Our study has revealed the existence of longer gonophores (up to 2.6 mm long) in *S.neglecta* than previously documented (Peruzzi and Carta 2013).

**Table 2.** Morphological comparison of *Silenemartinolii*, *S.neglecta* and *S.nocturna*. Quantitative values are expressed as 10–90 percentile intervals, with minimum and/or maximum in brackets and as mean ± standard deviation.

	*S.martinolii*	*S.neglecta*	*S.nocturna*
Ratio length/width of longest stem leaf	(2.11)2.20–3.10(3.11) 2.60 ± 0.43	(1.66)1.71–4,21(5.55) 2.92 ± 1.00	(1.05)3.06–9.13(11.0) 5.77 ± 2.42
Length of longest eglandular hair on lowest part of stem (mm)	(2.14)2.20–2.65(2.75) 2.42 ± 0.23	(1.95)2.05–2.80(3.10) 2.38 ± 0.31	(0.22)0.31–2.03(2.53) 1.09 ± 0.64
Length of longest eglandular hair on middle part of stem (mm)	1.20–1.67(1.78) 1.44 ± 0.24	(0.70)0.84–1.64(1.80) 1.21 ± 0.32	0.20–0.42(0.61) 0.28 ± 0.10
Length of longest glandular hair on middle part of stem (mm)	(0.43)0.53–0.97(1.02) 0.75 ± 0.22	(0.32)0.49–1.0(1.15) 0.68 ± 0.19	(0.14)0.17–0.22(0.23) 0.19 ± 0.02
Length of longest eglandular hairs on inflorescence axis (mm)	(0.35)0.37–0.55(0.58) 0.49 ± 0.09	(0.25)0.30–0.74(1.10) 0.51 ± 0.19	(0.14)0.18–0.28(0.42) 0.21 ± 0.05
Length of longest glandular hairs on inflorescence axis (mm)	(0.68)0.72–0.82(0.84) 0.78 ± 0.06	(0.57)0.60–0.83(1.20) 0.74 ± 0.13	(0.14)0.18–0.23(0.30) 0.20 ± 0.02
Ratio number of flowers/cm	(0.37)0.45–0.78(0.86) 0.62 ± 0.17	(0.30)0.45–1.0(1.09) 0.68 ± 0.18	(0.30)0.43–0.84(1.38) 0.64 ± 0.21
Calyx length of lower flower (mm)	(10.50)10.70–11.54(11.70) 11.10 ± 0.44	(10.3)10.5–11.62(12.0) 11.0 ± 0.46	(8.0)10.44–12.6(12.6) 11.12 ± 0.83
Longest calyx tooth length (mm)	(2.50)2.58–2.92(3.0) 2.76 ± 0.18	(2.5)2.7–3.14(3.4) 2.95 ± 0.21	(1.5)1.7–2.3(2.4) 2.0 ± 0.20
Longest calyx tooth width (mm)	1.60–1.70 1.64 ± 0.05	(1.0)1.36–2.0(2.1) 1.7 ± 0.28	(1.1)1.5–1.82(2.2) 1.68 ± 0.21
Ratio calyx length/calyx tooth	3.90–4.15(4.20) 4.02 ± 0.12	(3.38)3.50–3.98(4.12) 3.73 ± 0.19	(4.77)5.16–6.24(6.76) 5.59 ± 0.46
Length of longest eglandular hairs on calyx limb (mm)	(0.24)0.26–0.78(0.90) 0.49 ± 0.26	(0.18)0.21–0.69(0.88) 0.44 ± 0.20	(0.20)0.21–0.31(0.51) 0.25 ± 0.06
Length of longest glandular hairs on calyx limb (mm)	(0.50)0.58–0.77(0.78) 0.70 ± 0.11	(0.30)0.31–0.70(0.71) 0.55 ± 0.14	< 0.1
Length of longest eglandular hairs on calyx veins (mm)	(0.43)0.48–1.04(1.22) 0.72 ± 0.31	0.3–1.1(2.0) 0.83 ± 0.41	(0.20)0.22–0.76(0.92) 0.46 ± 0.21
Length of longest glandular hairs on calyx veins (mm)	(0.72)0.76–0.92(0.93) 0.85 ± 0.09	(0.27)0.39–1.16(1.97) 0.80 ± 0.27	0.10–0.14(0.15) 0.11 ± 0.02
Dominating type of hairs on calyx	glandular or eglandular + glandular	glandular or eglandular + glandular	eglandular
Petal limb incision	< 30% of limb length	< 30% of limb length	> 50% of limb length
Number of hairy stamen filaments	5–10	5–10	0
Gonophore length (mm)	2.0–2.26(2.3) 2.14 ± 0.13	2.0–2.34(2.6) 2.2 ± 0.15	(0.8)1.0–1.4(1.5) 1.18 ± 0.14
Largest seed length (mm)	(0.92)0.93–1.01(1.03) 0.97 ± 0.04	(0.90)0.91–1.02(1.05) 0.96 ± 0.04	(0.65)0.69–0.77(0.81) 0.72 ± 0.04
Largest seed width (mm)	(0.78)0.79–0.87(0.88) 0.83 ± 0.04	(0.76)0.80–0.89(0.91) 0.83 ± 0.04	(0.55)0.58–0.68(0.70) 0.61 ± 0.04

#### Calyx

As noted by Peruzzi and Carta (2013), *Sileneneglecta* is distinct from *S.nocturna* by its larger calyx and larger calyx teeth. Our study reveals that there is a wide overlap in the calyx length (Table 2), so this character does not allow an unequivocal separation of both taxa. Bocchieri and Mulas (1988) attributed a higher maximum value of calyx length to *S.martinolii* (9–13 mm). However, we have observed specimens of *S.neglecta* from Spain that also reach 13 mm in length (this measurement refers to calyces that do not correspond to the lowermost flower). The length of the calyces and calyx teeth of the lectotype of *S.mutabilis* is 8.8–11.2 mm and 1.4–2.0 mm, respectively.

**Figure 4.**
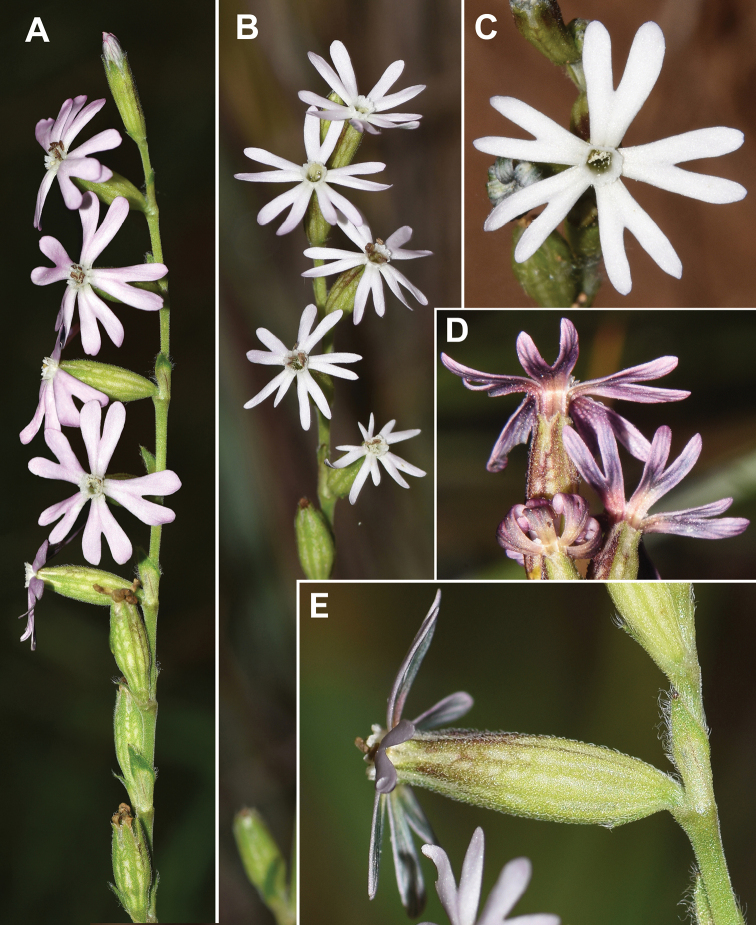
Field photos of *Silenenocturna***A, B** inflorescences **C, D** flowers **E** flower in lateral view. Spain, Sant Feliu de Codines, 29 May 2021, L. Sáez (herb. pers.).

**Figure 5.**
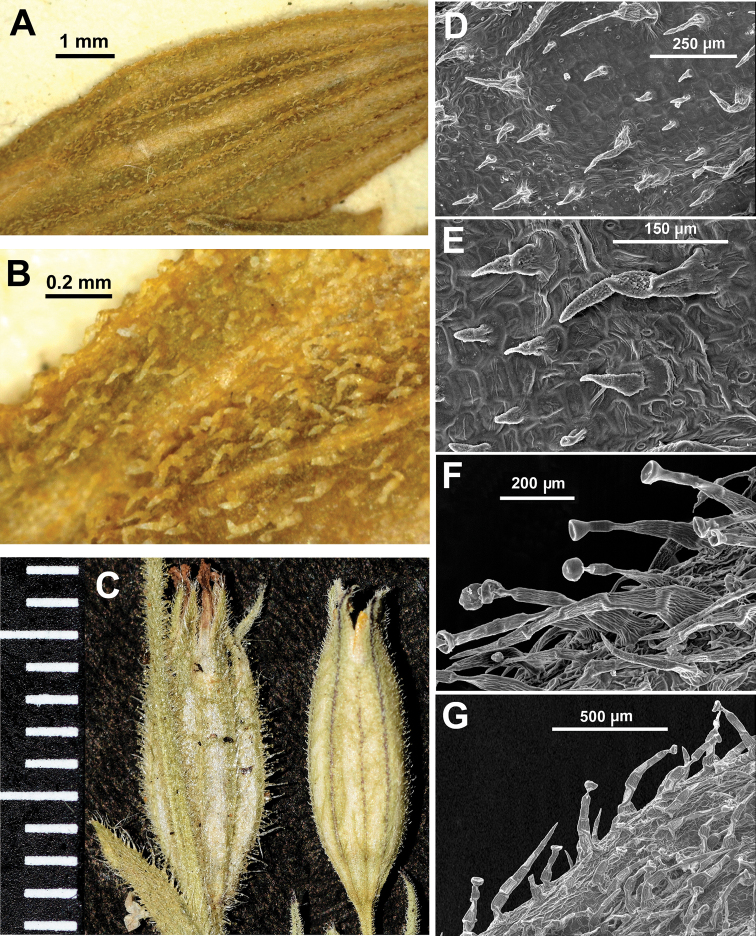
**A, B** indumentum of the lectotype of *Silenemutabilis* (LINN 583.8) **C** calyces of *S.neglecta* (left, Isola d’Elba, PI) and *S.nocturna* (Livorno, PI) **D, E** calyx with eglandular hairs of *S.nocturna* (**D** Tunisia, L. Sáez herb. pers. **E** Livorno PI) **F, G** calyx with glandular and eglandular hairs of *S.neglecta*. (Tunisia, Tabarka, L. Sáez herb. pers.).

The hairiness type of the calyx has taxonomic value. Two types of hairs were identified: eglandular and glandular hairs. The eglandular hairs (unicellular or pluricellular, the latter with up to nine cells) are progressively tapering towards the apex. These hairs show striated or verruculate walls. The eglandular hairs are usually short and antrorse in *Silenenocturna* and in the lectotype of *S.mutabilis*. These hairs are somewhat longer on the veins of the calyx (Table 2), whereas between the veins, they rarely exceed 0.2–0.3 mm in length (Table 2, Figs 4–5). The hairs of the calyx of the lectotype of *S.mutabilis* are eglandular, short (up to 0.2 mm long) and antrorse (Fig. 5). This morphology matches the typical calyx hairiness of *S.nocturna*. In *S.neglecta* and *S.martinolii*, the eglandular hairs are longer, patent or antrorse. These hairs are somewhat longer on the veins of the calyx if compared to those located between the veins (Table 2).

The glandular hairs are formed by a gland and stalk consisting of 1–8 cells. These glandular hairs show striated walls. The glandular hairs are found in *Sileneneglecta* and *S.martinolii*, while they are usually absent in *S.nocturna* and the type material of *S.mutabilis*. Very rarely, as was noted by Talavera (1990), very short glandular hairs can be observed in some specimens of *S.nocturna* (when occurring, a stalk consisting of 1–2 cells). Within *S.neglecta*, the hairiness of the calyces is somewhat variable. Some populations from Spain (Gavà, Bruguers, can Riera, L. Sáez, herb. pers.), Italy (Vulcano, L. Sáez, herb. pers.) have calyces covered with exclusively or mostly glandular patent hairs. In Tunisia (Tabarka, L. Sáez, herb. pers.) and southern Italy [Torregaveta (MA), Vigneti del Vesuvio (BC) and the type material of *S.neglecta*], the calyces are usually densely covered by eglandular hairs mixed with sparse glandular hairs. However, plants with both main types of calyx indument can be found within a single location [Italy, Isola Elba (PI); Tunisia: Tabarka (L. Sáez, herb. pers.)].

#### Corolla

Flower opening in *Silenenocturna* is mainly nocturnal, while in *S.neglecta* and *S.martinolii*, it is diurnal. The petals of *S.nocturna* and *S.mutabilis* are bifid (see also Linnaeus, 1753; 1756). In cleistogamous variants of *S.nocturna*, the petal limbs are very short (usually included) or even absent. On the contrary, the limb of the petals of *S.neglecta* and *S.martinolii* is subentire to emarginate (Fig. 3); the sinus of the limb can reach almost a third of its length. The lobes of *S.neglecta* and *S.martinolii*, when present, are much wider than those of *S.nocturna*, which are narrowly oblong to sublinear. After the description of *S.neglecta*, Tenore (1838) provided detailed illustrations of the species, showing the presence of pink petals with broad, not bifid limbs. The study of the lectotype of *S.neglecta* reveals that the limb of the petals is emarginate.

The colour of the corolla of *Sileneneglecta* and *S.nocturna* varies from pale pink (rarely white) to pinkish-purple. The petal colouration is variable within *S.nocturna*, even in the same population. The petals can be white (sometimes greenish in the abaxial surface), pale rose and even tinged with pink-purple (mainly on the abaxial side). This is remarkable, since this colouration was invoked by Linnaeus (1756) to describe his *S.mutabilis* (“*petalis post florescentiam extus purpurescentibus*”). We have observed specimens in populations from Spain (Barcelona Province) with this colouration attributed by Linnaeus to *S.mutabilis* and that perfectly fits the current concept of *S.nocturna* (Fig. 4).

#### Stamens

The hairiness of the stamen filaments is of taxonomic significance and, based on our observations, it is always related to the calyx hairiness (see above). The filaments are glabrous in *Silenenocturna*, whereas in *S.neglecta* and *S.martinolii*, alternate filaments are hairy at base (sometimes all are hairy at base). In those specimens in which all the stamen filaments are hairy (plants from Tabarka, Corona su Crabi, Torre gaveta, Campania and Volcano), the hairy portion of the filament is noticeably shorter in those stamens adjacent to the petals. Possibly, the difficult observation of this character can explain why it has gone unnoticed so far.

#### Seeds

**Figure 6.**
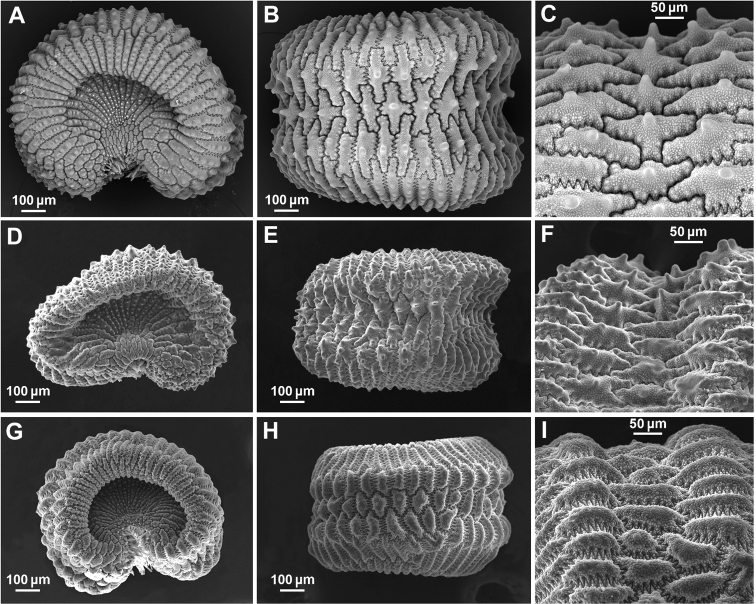
Seed micromorphology for *Sileneneglecta* from Spain, Gavà, Roca de Barret (L. Sáez herb. pers.) (**A, B, C**), Tunisia, Tabarka (L. Sáez herb. pers.) (**D, E, F**) and *S.nocturna* Italy, Livorno (PI) (**G, H, I**). Lateral view (**A, D, G**); dorsal view (**B, E, H**); dorsal furrow (**C, F, I**).

The seeds are reniform with excavate lateral faces and a dorsal furrow. The seeds of *Silenenocturna* are somewhat smaller than those of *S.neglecta* and *S.martinolii*. There are differences in colouration (grey or greyish-brown in *S.nocturna*, blackish to dark-brown in *S.neglecta* and *S.martinolii*). Peruzzi et al. (2014) stated that the lectotype of *S.mutabilis* has polygonal (star-shaped) dorsal cells. However, based on our observations, *S.neglecta* has elongate (more or less polygonal), not star-shaped dorsal cells (Fig. 6). The surface of the dorsal cells of *S.neglecta* has a more or less prominent central tubercle (up to 30 µm long), while in *S.nocturna*, these tubercles, when present, are not very prominent (less than 15 µm long). The pattern of morphological variability of *S.nocturna* seeds is complex and requires further study.

### ﻿Taxonomic treatment

After a critical macro- and micromorphological analysis and detailed studies of the protologues, we conclude that *Silenemutabilis* is not conspecific with *S.neglecta*. *S.mutabilis* shows characters which fall within the range of morphological variation of the currently recognised *S.nocturna*. Further research is needed to identify the existence of taxonomic units within *S.nocturna*, which could be a polyphyletic species. Our study shows that *S.neglecta* and *S.nocturna* are distinct, based on macro-features (leaf shape and petal limb shape) and micromorphological characters (calyx and stamens indumentum and SEM analysis of seeds). An amended description is here also provided for *S.neglecta*, based on herbarium and live specimens collected from North Africa, Italy, Sardinia, Sicily and Spain. Based on this macro- and micromorphological evidence, in addition to morphometric analysis, we can conclude that *S.martinolii* falls within the variation of *S.neglecta*. Our preliminary phylogenetic results (Mesbah et al., in prep.) suggest that *S.neglecta* is not related to *S.nocturna*, but more closely related to *S.gallica* L., the type species of the genus *Silene*.

#### ﻿ Silene neglecta

Taxon classificationPlantaeCaryophyllalesCaryophyllaceae

Ten., Fl. Neapol. Prodr. App. 5: 13. 1826.

BF0586CC-7EA9-5681-85B5-D111168D6F0B

≡ Silenenocturnasubsp.neglecta (Ten.) Arcang. Comp. Fl. Ital.: 88. 1882. Lectotype (designated by Peruzzi & Carta, 2013: 45): [Italy] Campania: Persano, s.d., *Tenore s.n.* (NAP!).
= Silenemartinolii Bocchieri & B.Mulas, Boll. Soc. Sarda Sci. Nat. 26: 301. 1988; syn. nov. Holotype (Bocchieri & Mulas, 1988: 301): [Italy. Sardinia] Isola il Toro, 22 May 1988, *Bocchieri & Mulas s.n.* (CAG!).

##### Description.

Annual herb (rarely biennial) green or greyish-green, hairy. Stems (10–)15–50(–70) cm, more or less erect (rarely prostrate-ascending), unbranched to much-branched, densely hairy, usually viscid above. Lower leaves spatulate to obovate; cauline leaves obovate to linear-lanceolate. Flowers 2–9(–10) in raceme-like monochasial cymes; lowermost pedicels shorter or longer than calyx (0.45–2.00 times as long as calyx in fruit), erect to patent, pubescent-glandular. Calyx 8–13 mm long, subcylindrical to cylindrical-ovoid in flower, attenuate, becoming subovoid to ovoid in fruit, usually densely hairy, covered by long eglandular hairs (up to 1.9 mm long) and glandular hairs (up to 2 mm long); calyx teeth triangular to linear-lanceolate or linear, acute; veins anastomosing. Petals pale pink to pinkish-purple, rarely white; limb 4.5–6.0 mm, subentire or emarginate; coronal scales 1.2–2.2 mm, whitish to pink. Stamens with alternate filaments hairy at base, sometimes all hairy at base; anthers 1.0–1.5 mm long, lilac to purple, exserted from corolla mouth. Gonophore (1.8–)2.0–2.4(–2.6) mm long, puberulent. Capsule 6.0–8.5 mm, subcylindrical, enclosed within the calyx. Seeds (0.90–)0.91–1.02(–1.05) × (0.76–)0.80–0.89(–0.91) blackish to dark brown; faces deeply concave, tuberculate; back wide, slightly canaliculate.

##### Flowering time.

Between March (exceptionally at the beginning of February) and May.

##### Chromosome number.

2*n* = 24 (Peruzzi and Carta 2013; Bosch et al. 2019).

##### Habitat.

Rocky places, maritime sands and grassland, usually on siliceous substrata, 0–1700 m a.s.l.

##### Distribution.

Central and southern Italy, south-western Sardinia, Sicily, northern Algeria and Tunisia and north-eastern Spain.

##### Specimens examined.

**Algeria**: Terrains siliceux, berges maritimes sur la route de Cap de Garde, au-dessous de la colline des Caroubiers à Bône, 5 March 1867, *Tribout* (P05032571); Bône, rochers maritimes, 12 Apr 1867, *Tribout* (P05033844); subéraire, près de Bessombourg, 500–600 m alt., Kabylie de Collo, 17 May 1944, *L. Faurel* (P05110031, P05135518); subéraire, des crètes de Boudra, près de Bessombourg, 600 m alt., Kabylie de Collo, 26 May 1944, *L. Faurel* (P05050465); pentes rocheuses du flanc sud du Djebel Tababort, 1700 m alt., (Dt. de Constantine), 29 May 1952, *L. Faurel* (P05110033); près du Col de Terras, 800 m alt., environ 5 km à l’ouest de Zitouna (ex Bessombourg), Kabylie de Collo, 8 Juin 1984, *A. Dubuis* (MPU286766); **Italy**: Campania: Ischia, Oct 1835, *Gussone* (K00728586); Campania, vigneti del Vesuvio, 150 m alt., 29 Apr 1911, *G. Pellanda* (BC8940, MA 31093, US1272504); Campi Flegrei, prope Capo Miseno, 100 m alt., solo siliceo, 15 May 1913, *M. Guadagno* (BC8939); Torre Gaveta, May 1913, *M. Guadagno* (MA31092, MA31096, MPU300592); Sardinia: Isola il Toro, 22 May 1988, *E. Bocchieri & B. Mulas* (CAG, holotype *S.martinolii*); Isola Rossa, Golfo di Teulada, 10 July 1988, *E. Bocchieri* (CAG, sub *S.martinolii*); Isola la Vacca, 18 Feb 1990, *E. Bocchieri* (CAG, sub *S.martinolii*); S’Aqua sa Canna, Isola di S. Antioco, 12 Apr 1992, *L. Mossa* (CAG, sub *S.martinolii*); Sa Corona su Crabi, isola di S. Antioco, 20 May 1993, *L. Mossa* (CAG, sub *S.martinolii*); Sicily: Lipari, Apr 1902, *Ross 317* (WAG); Vulcano Island, Apr 2017, *D. Carrera* (grown from seed by J. López & L. Sáez, May 2018, L. Sáez, herb. pers.); Tuscany: Insula Igilium (Giglio), 17 May 1894 *Sommier* (MPU772254); Insula Igilium (hod. Giglio), prope Portum, 5 Apr 1899, *Sommier* (MA31097); Insula Inarime, in campis aridis, May 1905, *M. Guadagno* (US548489); Isola d’Elba (Tuscan Archipelago), Miniera del Ginevro, 27 Apr 2017, *B. Pierini* (PI, sub *S.mutabilis*). **Spain**: Barcelona: Bruguers, 25 May 1984, *C. Blanché* (BCN 41111); Garraf massif, Gavà, Ermita de Bruguers, siliceous rocks, 265 m alt., 6 May 2015, *L. Sáez LS-7626* (L. Sáez, herb. pers.); Cervelló, Can Riera, 350 m alt., 9 May 2015, *D. Carrera & L. Sáez LS-7629* (L. Sáez, herb. pers.); Torrelles de Llobregat, Roca Plana NE from Turó de la Bruguera, 280 m alt., 23 Apr 2016, *L. Sáez LS-7706* (L. Sáez, herb. pers.); Gavà, Roca del Barret, 23 Apr 2016, *L. Sáez* (L. Sáez, herb. pers.); ibidem, 26 May 2016, *L. Sáez LS-7707* (L. Sáez, herb. pers.); ibidem, 29 Apr 2021, *L. Sáez* (herb. pers.); between castell d’Eramprunyà and Bruguers, 26 May 2016, *L. Sáez* (herb. pers.). **Tunisia**: Tabarka, maritime sands, March 2017, *R. El Mokni* (L. Sáez, herb. pers.; R. El Mokni, herb. pers.).

#### ﻿ Silene nocturna

Taxon classificationPlantaeCaryophyllalesCaryophyllaceae

L.

F4651A48-134F-5C79-ABCA-FDD3E52DA392

##### Specimens examined.

**Algeria**: Batna: Campus Batna II, 14 Apr 2019, *F. Bekdouche* (GB); Bouira: Crête Rouge, 23 Apr 2017, *M. Mesbah* (ENSA); Bechloul devant la porte du lycée (Bouira), 17 Dec 2019, *M. Mesbah* (GB); W Sétif, a 800 m de Tizi N'Bechar, Apr 2019 *F. Bekdouche* (GB); Tlemcen, May 2018, *B. Babali* (ENSA); forèt de Remchi - Tlemcen, 1 June 2018, *B. Babali* (GB); Bejaia, Melbou 16 Apr 2019, *M. Mesbah* (GB). **Croatia**: Starigrad, Paklenica, 2 m alt., 27 Apr 2013, *L. Peruzzi* (PI). **France**: Bouches du Rhône: Marseille, 9 May 1866, *Linder* (BC135145); Garlaban, 19 May 1971, *R. Roncart* (BC803193); Corsica: Corse-du-Sud: Bonifacio, sémaphore de Pertusato, along the path, 85 m alt., sandy beach, 28 Apr 2008, *B. Frajman & P. Schönswetter* (GB); Hérault: Maguelone, Montpellier, lungo la strada che porta alla cathédrale Saint-Pierre-et-Saint-Paul de Maguelone, 2 m alt., 11 June 2016, *F. Roma-Marzio 379/2, G. Astuti, M. D’Antraccoli & L. Peruzzi* (PI); **Greece**: Nomos Fokidas, About 5 km W Galaxidia, 5 May 1984 *B. Oxelman & L. Tollsten* (GB); Nomos Arkadias, Mount Parnon, 600–700 m alt., 29 Apr 1985, *B. Oxelman & L. Tollsten* (GB); Delfi, roadside in Parnassidos Province of Fokidos, 6 May 1984, *B. Oxelman* (GB-0194077). **Iran**: Gilan Province, *Mozaffarian* (TUH6771). **Italy**: Basilicata: Potenza in pascuis, 50–850 m alt., 13 May 1928, *Gavioli* (BC8926); Calabria: Tarsia (Cosenza), 156 m alt., May 2017, *G. Fasano* (PI, sub *S.mutabilis*); Campania: Parco Archeologico di Carbonara, Aquilonia, 600–675 m alt., 3 June 2015, *G. Astuti, L. Peruzzi & F. Roma-Marzio* (PI); Tuscany: Monte Pisano, Annunziata, 25 m alt., 11 May 2010, *L. Peruzzi, B. Pierini & G. Bedini* (PI); Isola di Capraia, 32TNN67.67, 242 m alt., 1 Apr 2012 *L. Peruzzi & G. Gestri* (PI); Rosignano Marittimo (Livorno), Castiglionello, 15 June 2016, *L. Peruzzi* (PI, sub *S.mutabilis*). **Morocco**: Tangier-Tetouan: pr. Zinnat, inter Tandja & Tetouan, 28 May 1930, *Font Quer* (BC110896). **Spain**: Balearic Islands: Cabrera, camino de sa Vicaria, 27 July 1947, *Palau Ferrer* (MA31038); Cabrera, Es Penyal Blanc, 3 Apr 1948, *P. Ferrer* (BC104070); Formentera, La Mola, 1918, *Gros* (BC110894); Eivissa, Santa Eulàlia, 2 June 1918, *Gros* (BC 110897); Pla de Vila, 15 May 1919, *Font Quer* (BC110902); Mallorca, Bellver, pr. Palma, 12 May 1920, *Font Quer* (BC110901); Mallorca, Lluc, Clot d’Albarca, s.d., [Bonafè] (HJBS 1243); Mallorca, Palma towards Secar de la Real, 45 m alt., 2 June 2013, *L. Sáez, LS-7382* (L. Sáez, herb. pers.); Menorca, Ciutadella, Montefi, en el km 43 de la carretera, 31TEE7328, 40 m alt., 30 Apr 1951, *P. Montserrat* (JACA36851); Ciutadella, Torre del Ram, 31TEE6929, 40 m alt., 16 Apr 1957, *P. Montserrat* (JACA297519); Menorca, Trabaluger, bajando de Son Olivar, 60–70 m alt., 22 Apr 1957, *P. Montserrat* (JACA297520, JACA 297521); Menorca, Algaiarens, 23 Apr 1993, *P. Fraga* (L. Sáez, herb. pers.); Menorca, Arenal de Salomó, 11 June 1993, *P. Fraga* (L. Sáez, herb. pers.); Barcelona: Hospitalet de Llobregat, 26 Apr 1872, *A.C. Costa* (BC614253); Maresme, Camí Geganta, 7 Apr 1936, *P. Montserrat* (BC609200); Turó de Santa Margarida, Cànoves i Samalús, 350 m alt., 11 Apr 2015, *L. Sáez LS-7604* (L. Sáez, herb. pers.); Sant Feliu de Codines, towards Sot de l’Ullar, 460 m alt., grassland, siliceous rocks, 22 Apr 2016, *L. Sáez* (L. Sáez, herb. pers.); Viladecans, davant Parc de Can Guardiola, 31TDF1574, 3 May 2021, *A. Salvat* (L. Sáez, herb. pers.); Sant Feliu de Codines, Roques d’en Pere Pericó, 550 m alt., 29 May 2021, *L. Sáez LS-7910* (L. Sáez, herb. pers.); Castelló: Cami de Villareal, May 1954, *M. Calduch* (BC128966); Girona Province, Sant Julià de Llor, 31 May 1920, s.r. (BC8927); Jaén: Albandes, márgenes del camino a Torres, 7 June 1925, *Cuatrecasas* (BC8897); Huesca: Salto de Roldan, YM1581, 1000 m alt., 19 July 1980, *J.M. Martí* (BC922231); La Almunia del Romeral, YM2476, 620 m alt., 3 May 1981, *J.M. Montserrat* (BC922230 sub S.nocturnasubsp.neglecta); Málaga: plaza Adnana, waste land, 15 Apr 1968, *Strandhede & al. 45* (GB); Navarra: Unzue, Puerto del Carrascal, 590 m alt., 22 May 1988, *I. Aizuru & P. Catalán* (BC834596); Tarragona: Coll de Balaguer, Hospitalet de l’Infant, 27 Apr 1974, *R. Folch & E. Velasco* (BC627072). **Tunisia**: Bizerta, 5 March 2017, *R. El Mokni* (L. Sáez, herb. pers.; R. El Mokni, herb. pers.); Melloula, 7 March 2017, *R. El Mokni* (L. Sáez, herb. pers.; R. El Mokni, herb. pers.); Monastir, 25 March 2017, *R. El Mokni* (L. Sáez, herb. pers.; R. El Mokni, herb. pers.); Tabarka, 7 March 2017, *R. El Mokni* (L. Sáez, herb. pers.; R. El Mokni, herb. pers.); Tabarka, Kroumiria, 1 May 2018, *R. El Mokni* (L. Sáez, herb. pers.; R. El Mokni, herb. pers.). **Turkey**: Mugla: road Mugla-Marmaris, 12 km N of Marmaris, 4 May 1988, *B. Oxelman* (GB). **Unknown origin**: Herb. Linn. No. 583.8 (LINN; lectotype).

## Supplementary Material

XML Treatment for Silene neglecta

XML Treatment for Silene nocturna

## ﻿Appendix I

List of specimens included in morphometric analyses.

*S.martinolii*

**Italy**: Sardinia, Isola il Toro, 22 May 1988, *E. Bocchieri & B. Mulas* (CAG); Isola Rossa, Golfo di Teulada, 10 July 1988, *E. Bocchieri* (CAG); S’Aqua sa Canna, Isola di S. Antioco, 12 Apr 1992, *L. Mossa* (CAG) [2 specimens]; Sa Corona su Crabi, Isola di S. Antioco, 20 May 1993, *L. Mossa* (CAG).

*S.neglecta*

**Italy**: Insula Igilium (hod. Giglio), prope Portum, 5 Apr 1899, *S. Sommier* (MA 31097); Campania, vigneti del Vesuvio, 150 m alt., 29 Apr 1911, *G. Pellanda* (BC 8940, MA 31093) [2 specimens]; Campi Flegrei, prope Capo Miseno, 100 m alt., solo siliceo, 15 May 1913, *M. Guadagno* (BC8939); Torre Gaveta, May 1913, *M. Guadagno* (MA 31092, MA 31096) [2 specimens]; Italy, Isola d’Elba (Tuscan Archipelago), Miniera del Ginevro, 27 Apr 2017, *B. Pierini* (PI) [3 specimens]; Sicily, Vulcano Island, Vulcano Island, April 2017, *D. Carrera* (grown from seed by J. López & L. Sáez, May 2018, L. Sáez, herb. pers.) [2 specimens]. **Spain**: Barcelona Province, Cervelló, Can Riera, 350 m alt., 9 May 2015, *D. Carrera & L. Sáez LS-7629* (L. Sáez, herb. pers.); Torrelles de Llobregat, Roca Plana NE from Turó de la Bruguera, 280 m alt., 23 Apr 2016, *L. Sáez LS-7706* (L. Sáez, herb. pers.); Gavà, Roca del Barret, 23 Apr 2016, *L. Sáez* (L. Sáez, herb. pers.) [4 specimens]; ibidem, 26 May 2016, *L. Sáez* (L. Sáez, herb. pers.); between castell d’Eramprunyà and Bruguers, 26 May 2016, *L. Sáez* (herb. pers.) [2 specimens]. **Tunisia**: Tabarka, maritime sands, March 2017, *R. El Mokni* (L. Sáez, herb. pers.; R. El Mokni, herb. Pers.) [4 specimens]; Tabarka, Kroumiria, 1 May 2018, *R. El Mokni* (L. Sáez, herb. pers.; R. El Mokni, herb. pers.) [3 specimens].

*S.nocturna*

**Algérie**: Crête Rouge, 1 June 2017, *M. Mesbah* (GB); forèt de Remchi - Tlemcen, 1 June 2018, *B. Babali* (GB); Campus Batna II (Batna), 14 Apr 2019, *F. Bekdouche* (GB); Melbou (Bejaia), 16 Apr 2019, *M. Mesbah* (GB); A 800 m de Tizi N. Bechar (W Sétif), Apr 2019, *F. Bekdouche* (GB); Bechloul devant la porte du lycée (Bouira), 17 Dec 2019, *M. Mesbah* (GB). **Croatia** : Starigrad, Paklenica, 2 m alt., 27 Apr 2013, *L. Peruzzi* (PI). **France** : Marseille, 9 Mai 1866, *Linder* (BC135145); Bouches du Rhône, Garlaban, 19 May 1971, *R. Roncart* (BC803193); Corsica, Corse-du-Sud: Bonifacio, sémaphore de Pertusato, along the path, 85 m alt., sandy beach, 28 Apr 2008, *B. Frajman & P. Schönswetter* (GB); Maguelone, (Montpellier), lungo la strada che porta alla cathédrale Saint-Pierre-et-Saint-Paul de Maguelone, 2 m alt., 11 June 2016, *F. Roma-Marzio 379/2*, *G. Astuti*, *M. D*’*Antraccoli & L. Peruzzi* (PI). **Greece**: Nomos, Fokidas, About 5 km W Galaxidia, limestone cliffs, 5 May 1984, *B. Oxelman & L. Tollsten* (GB); Nomos, Arkadias, Mount Parnon, 600–700 m alt., 29 Apr 1985, *B. Oxelman & L. Tollsten* (GB); Delfi, roadside in Parnassidos Province of Fokidos, 6 May 1984, *B. Oxelman* (GB). **Italy**: Potenza in pascuis, 50–850 m alt., 13 May 1928, *Gavioli* (BC 8926); Monte Pisano, Annunziata, 25 m alt., 11 May 2010, *L. Peruzzi*, *B. Pierini & G. Bedini* (PI) [2 specimens]; Isola di Capraia, 32TNN67.67, 242 m alt., 1 Apr 2012 *L. Peruzzi & G. Gestri* (PI) [2 specimens]; Rosignano Marittimo (Livorno), Castiglionello, 15 June 2016, *L. Peruzzi* (PI, sub *S.mutabilis*) [2 specimens]; Calabria, Tarsia (Cosenza), 156 m alt., May 2017, *G. Fasano* (PI, sub *S.mutabilis*). **Spain**: Balearic Islands, Ciutadella de Menorca, Torre del Ram, 31TEE6929, 40 m alt., 16 Apr 1957, *P. Montserrat* (JACA297519); Menorca, Trabaluger, bajando de Son Olivar, 60–70 m alt., 22 Apr 1957, *P. Montserrat* (JACA297520); Menorca, Ciutadella, Montefi, en el km 43 de la carretera, 40 m alt., 30 Apr 1951, *P. Montserrat* (JACA36851); Mallorca, Palma towards Secar de la Real, 45 m alt., 2 June 2013, *L. Sáez*, *LS-7382* (L. Sáez, herb. pers.); Barcelona Province, Sant Feliu de Codines, towards Sot de l’Ullar, 460 m alt., 22 Apr 2016, *L. Sáez* (L. Sáez, herb. pers.); Sant Feliu de Codines, Roques d’en Pere Pericó, 550 m alt., 29 May 2021, *L. Sáez LS-7910* (L. Sáez, herb. pers.); Viladecans, davant Parc de Can Guardiola, 31TDF1574, 3 May 2021, *A. Salvat* (L. Sáez, herb. pers.); Jaén Province, Albandes, márgenes del camino a Torres, 7 June 1925, *Cuatrecasas* (BC8897); Huesca Province, La Almunia del Romeral, YM2476, 620 m alt., 3 May 1981, *J.M. Montserrat* (BC922230); Málaga Province, plaza Adnana, waste land, 15 Apr 1968, *Strandhede & al. 45* (GB); Navarra Province, Unzue, Puerto del Carrascal, 590 m alt., 22 May 1988, *I. Aizuru & P. Catalán* (BC834596); Tarragona Province, Coll de Balaguer, Hospitalet de l’Infant, 27 Apr 1974, *R. Folch & E. Velasco* (BC627072). **Tunisia**: Bizarte, 5 March 2017, *R. El Mokni* (L. Sáez, herb. pers.; R. El Mokni, herb. pers.); Melloula, 7 March 2017, *R. El Mokni* (L. Sáez, herb. pers.; R. El Mokni, herb. pers.). **Turkey**: Mugla: road Mugla-Marmaris, 12 km N of Marmaris, 4 May 1988, *B. Oxelman* (GB).
